# Coupling Single-Drop Microextraction with SERS: A Demonstration Using p-MBA on Gold Nanohole Array Substrate

**DOI:** 10.3390/s19204394

**Published:** 2019-10-11

**Authors:** Elias B. Santos, Chiara Valsecchi, Jaderson L. S. Gonçalves, Luis F. Ávila, Jacson W. Menezes

**Affiliations:** 1 LQANano, Federal University of São Paulo, São José dos Campos-SP, 12231-280, Brazil; santos.barros@unifesp.br; 2 Engineering Department, Federal University of Pampa, Alegrete-RS, 97546-550, Brazil; jadersg@alunos.unipampa.edu.br (J.L.S.G.); jacsonmenezes@unipampa.edu.br (J.W.M.); 3 Applied Optics Laboratory–School of Technology, State University of Campinas, Limeira–SP, 13484-350, Brazil; lfavila@ft.unicamp.br

**Keywords:** gold nanohole array, interference lithography, pre-concentration, Raman signature, low concentration detection

## Abstract

Single-drop microextraction (SDME) was coupled with surface-enhanced Raman scattering (SERS) to provide sample extraction and pre-concentration for detection of analyte at low concentrations. A gold nanohole array substrate (AuNHAS), fabricated by interference lithography, was used as SERS substrate and para-mercaptobenzoic acid (p-MBA) was tested as a probe molecule, in the concentration range 10^−8^–10^−4^ mol L^−1^. With this approach, a limit of 10^−7^ mol L^−1^ was clearly detected. To improve the detection to lower p-MBA concentration, as 10^−8^ mol L^−1^, the SDME technique was applied. The p-MBA Raman signature was detected in two performed extractions and its new concentration was determined to be ~4.6 × 10^−5^ mol L^−1^. This work showed that coupling SDME with SERS allowed a rapid (5 min) and efficient pre-concentration (from 10^−8^ mol L^−1^ to 10^−5^ mol L^−1^), detection, and quantification of the analyte of interest, proving to be an interesting analytical tool for SERS applications.

## Highlights:

- Gold nanoholes array substrate (AuNHAS) prepared by interference lithography is a reproducible substrate for surface-enhanced Raman scattering (SERS) analysis.
- AuNHAS was applied as SERS substrate to detect low concentration of p-MBA.
- Single-drop micro-extraction (SDME) is a pre-concentration technique for low concentration analyte.
- Coupling SDME with SERS made possible the detection of para-mercaptobenzoic acid (p-MBA) at nanomolar concentration.

## 1. Introduction

Surface-enhanced Raman scattering (SERS) has been widely applied in chemistry, environmental analyses, material science, and biosensors, among others, as an extremely sensitive and fast technique for analytical applications, including for single-molecule detection [1,2,3]. The Raman scattering enhancement mechanism is extremely surface dependent, and the success of SERS depends on several factors: Most importantly, the molecule Raman cross section, its surface affinity, and the plasmonic activity of the nanostructures used as substrate [1,2]. Thiophenol-based molecules, as *para*-mercaptobenzoic acid (p-MBA), present a strong binding affinity of the thiolate group with gold for chemical attachment on the substrate surface, as well as a strong Raman signal of the benzene ring; for these reasons, they are often used as probing molecules [4].

Moreover, several nanofabrication strategies have been explored for the fabrication of SERS substrates with enhanced electric fields (hot spots), and three main classes of SERS substrates have been developed: Metallic rough surfaces, colloidal nanoparticles, and periodic nanostructures [5]. A reproducible substrate is required to obtain an excellent homogeneity of hot spots, which means high reproducibility of the SERS signal and low interference from the substrate background [5,6]. Gold nanohole array substrates (AuNHAS) are very attractive for this purpose, since they are periodically structured. Gold nanohole arrays of different shapes, sizes, and periodicities have been fabricated by electron beam lithography (EBL) or focused ion beam (FIB), among other techniques, and successfully applied as substrates to monitor rhodamine 6G (Rh6G) dyes and other molecules [7,8]. Interference lithography (IL) was employed here as an alternative fabrication method that provides a convenient approach to generate a reproducible large area AuNHAS (1-inch squared) at lower costs in respect to common serial techniques, such as FIB and EBL [9]. In a general manner, for very low concentration analysis, i.e., below 10^−6^ mol L^−1^, many nanostructured materials may not be sensitive enough for an efficient detection. To overcome this, a pre-concentration technique, such as the single-drop microextraction (SDME), can be introduced as an analytical strategy to improve low concentration detection.

SDME is a liquid-phase microextraction technique, where a micro drop of an organic solvent is used to transfer a dissolved substance from one liquid phase to another liquid phase (immiscible or partially miscible) in contact with it [10]. SDME has become more popular than other microextraction techniques because it is simple, cost-effective, easy to operate, and almost solvent-free [10]. The technique has been employed successfully for trace analyses in environmental, food, and sensing applications [11,12,13] and it has also been coupled with various analytical techniques, such as gas chromatography (GC), high-pressure liquid chromatography (HPLC), and capillary electrophoresis-mass spectrometry (CE-MS), among others [14,15,16]. In most cases, couplings were made in off-line mode, except for the report of Kim et al. [16], where the authors showed a direct coupling of SDME to capillary electrophoresis-mass spectrometry. On the other hand, there are no reports, to our knowledge, about coupling of SDME with SERS.

In this context, we proposed the application of a single-drop microextraction technique as a pretreatment step before SERS analysis of low concentration analytes. For this purpose, p-MBA was used as a Raman probe molecule and gold nanohole array was used as SERS substrate. The off-line coupling of SDME with SERS was applied only when direct p-MBA detection was no longer possible.

## 2. Materials and Methods

### 2.1. Preparation of Gold Nanohole Array Substrates

**Figure 1:**
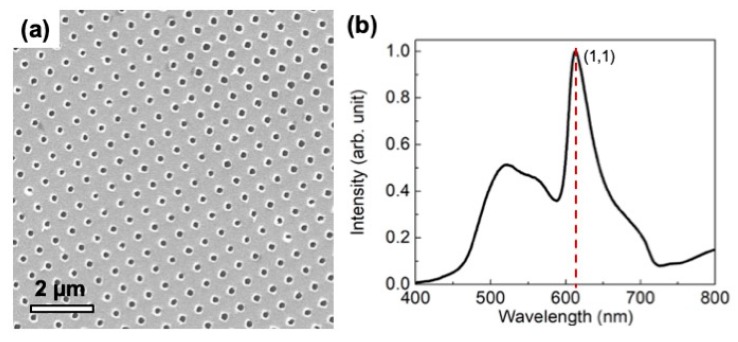
(**a**) SEM image and (**b**) transmission spectra of a gold nanohole array fabricated by interference lithography (IL) with a period of ʌ = 500 nm. The plasmonic peak of resonance order (1,1) presents a maximum at 615 nm.

The gold nanohole array substrates (AuNHAS), with period ʌ = 500 nm, were fabricated using a combination of two-beam interference lithography (IL) and a lift-off step. Details of the experimental process can be found in Menezes et al. [9]. Briefly: One portion of the wavefront of a spatially filtered laser beam (458 nm) impinged directly incident on the substrate surface, while the other portion was reflected by a mirror toward the sample. The interference pattern was generated by the superimposition of these two beams. The sample was coated with a positive 500-nm thick photosensitive material. The photoresist layer was exposed twice to the same interference fringe pattern, with a 90° rotation between exposures, to produce the two-dimensional template. After the exposures, with a dose of 200 mJ/cm^2^ each, the photoresist was developed for 50 s with the Microposit AZ 351 developer diluted 1:3 in water. The photoresist template was coated with an 80-nm gold film by e-beam evaporation, and then the photoresist was removed using acetone. The AuNHAS were characterized by FEG (field emission gun microscope, model Inspect) with 20 kV of acceleration voltage, and energy-dispersive X-ray spectroscopy (EDS) for elemental analysis; the sample presented a homogeneous gold surface and periodic holes over an area of 2 cm^2^, as can be seen in Figure 1a (showing an actual total area of ~10 μm^2^). Figure 1b reports the transmission spectra of the AuNHAS, showing the sharp plasmonic peak with resonance at 615 nm for the (1,1) order.

### 2.2. SERS Measurements

SERS spectra were acquired using a confocal Jobin-Yvon T64000 Raman spectrometer system, equipped with a liquid-N_2_-cooled charge-coupled device (CCD) detector. The excitation source was a He-Ne laser at 633 nm. The laser power at the sample surface was about 2.5 mW. The laser was focused with a 100x focal-lens objective, to obtain a diameter of 1 µm at the surface, for an exposure time of 10 s. All the measurements were done in triplicate. After that, aliquots of 25 μL from each solution of p-MBA were dropped onto the surface and dried. The surface Raman mapping data was carried out using the p-MBA solution of 10^−6^ mol L^−1^, collecting a total of 169 SERS spectra.

### 2.3. Single-Drop Microextraction of Para-Mercaptobenzoic Acid

**Figure 2:**
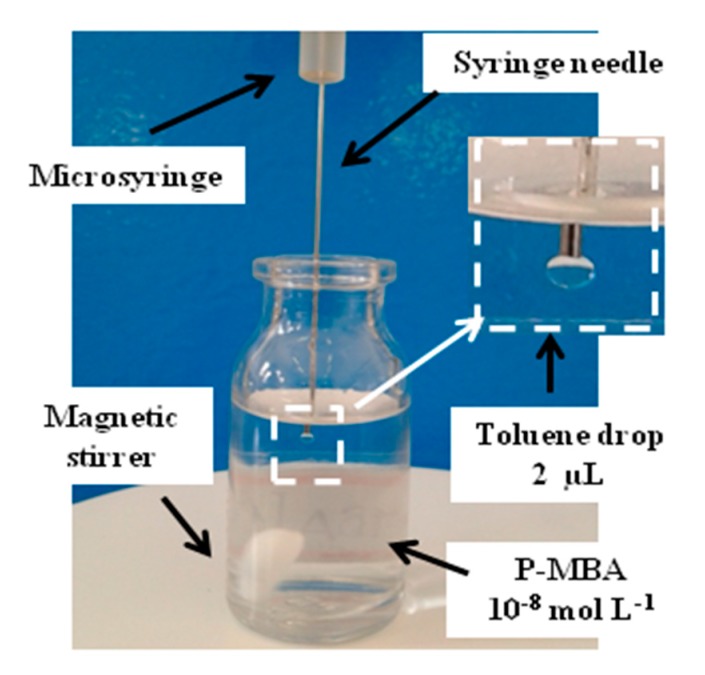
Single-drop microextraction (SDME) experimental setup for microextrations of *para*-mercaptobenzoic acid (*p*-MBA) from a 10^−8^ mol L^−1^ solution. In the inset, a detailed photo of the 2 μL drop extraction solvent (toluene).

The experimental setup shown in Figure 2 was applied for the SDME step. A 100-µL Hamilton microsyringe, containing 10 µL of toluene, was fixed on a platform and pressed carefully from the top in order to dip its needle into the glass vessel containing 3 mL of p-MBA aqueous solution, 10^−8^ mol L^−1^. A toluene drop of 2 μL was generated inside the p-MBA solution, which was kept at 22 °C and stirred at 100 rpm for 5 min. After this time, the drop was collected back and dropped onto the AuNHAS. The procedure was repeated one more time, dropping the extracted solution (p-MBA in toluene) on a different area of the same AuNHAS. After solvent evaporation, the SERS spectra were collected.

## 3. Results and Discussion

**Figure 3:**
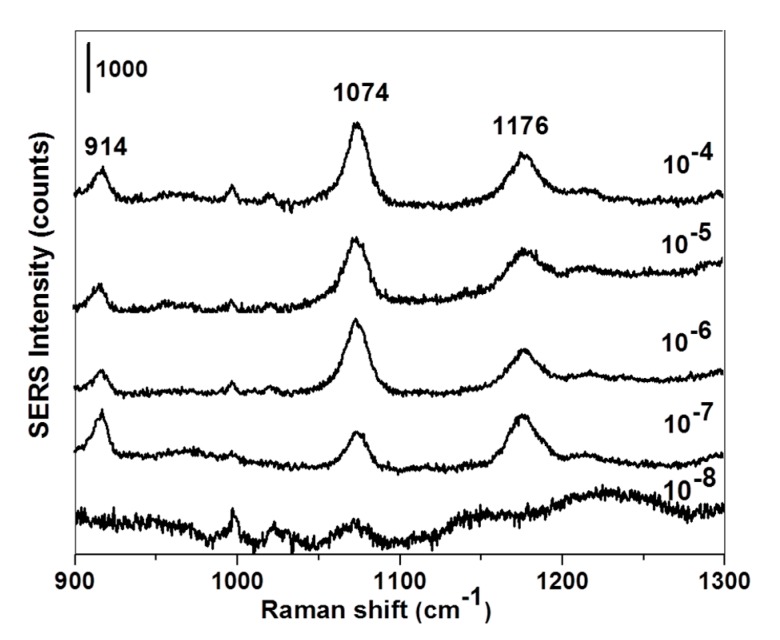
Representative surface-enhanced Raman scattering (SERS) spectra for p-MBA solutions at different concentrations (10^−8^–10^−4^ mol L^−1^). Laser wavelength: 633 nm; laser power: 2.5 mW. The spectra were normalized and the scale bar is 1000 count.

Thiophenol-based molecules, such as p-MBA, are a class of compounds commonly used as Raman probe for SERS studies due to the large Raman signal of the benzene ring, offering great sensitivity and specificity [4]. Figure 3 displays one representative SERS spectra for all the p-MBA concentrations used in the study. The peak at 914 cm^−1^ can be related to the δ(CSH) bending mode, and the band at 1176 cm^−1^ was associated with the δ(CH) deformation vibration. Moreover, the intensity of the band at 1074 cm^−1^, assigned to the p-MBA aromatic ring breathing [4], decreased regularly when the concentration changed. For this reason, the Raman signal at 1074 cm^−1^ was selected as an indicator for the SERS data. Moreover, in Figure 3, the p-MBA concentration of 10^−7^ mol L^−1^ was considered the lowest concentration with a distinctly detected SERS signal on the fabricated AuNHAS.

**Figure 4:**
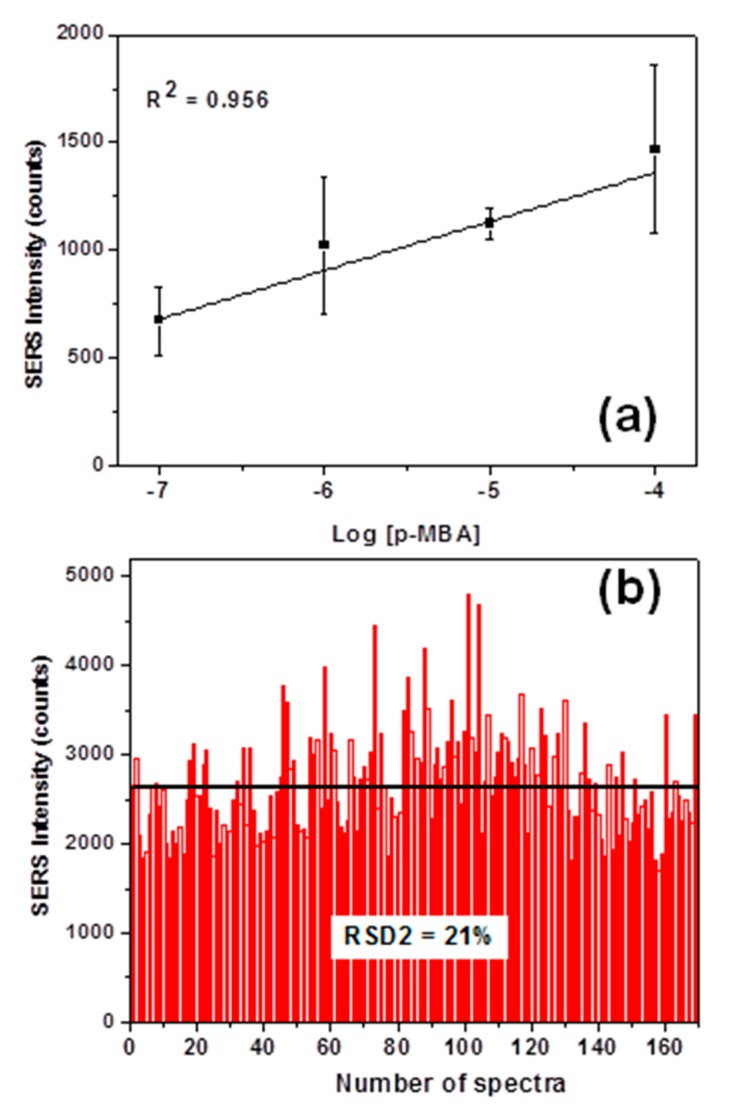
(**a**) Dependence of the average SERS intensity at 1074 cm^−1^, with the logarithm of the p-MBA concentrations. (**b**) SERS intensities distribution at 1074 cm^−1^ for the Raman mapping on AuNHAS of the 10^−6^ mol L^−1^ p-MBA solution. Laser wavelength: 633 nm; laser power: 2.5 mW.

In order to evaluate the reproducibility of the SERS data collected on our substrate, the average SERS intensities at 1074 cm^−1^ for each p-MBA concentration were analyzed (Figure 4a) together with the intensity distribution at 1074 cm^−1^ from 169 spectra (SERS mapping, Figure 4b).

Figure 4a demonstrates a good correlation between the average SERS intensities at 1074 cm^−1^ against the logarithm of p-MBA concentrations (10^−7^ to 10^−4^ mol L^−1^), leading to a coefficient of determination (R-squared) of 0.956. This result suggests that the SERS intensities are proportional to the molecular amount of p-MBA on AuNHAS. A similar result was reported by Saleh et al. [17]; therefore, the relationship obtained can also be used as a calibration curve for future SERS quantitative analysis of p-MBA on AuNHAS. In Figure 4b, the intensity of 1074 cm^−1^ band from the 169 SERS spectra of the Raman mapping is plotted as a histogram: The black line in Figure 4 represents the average intensity value detected of 2671 ± 568 counts. The spot-to-spot variation of the band intensities was mainly caused by the differences in the localized surface plasmon resonance (LSPR), as this electromagnetic field enhancement was the major contribution toward SERS [18]. On our AuNHAS, a relative standard deviation (RSD) of 21% was achieved, indicating a good signal reproducibility, as other substrates based on LSPR reported RSD between 2030% [18], and other good reproducible substrates presented values of slightly less than 20% [19].

**Figure 5:**
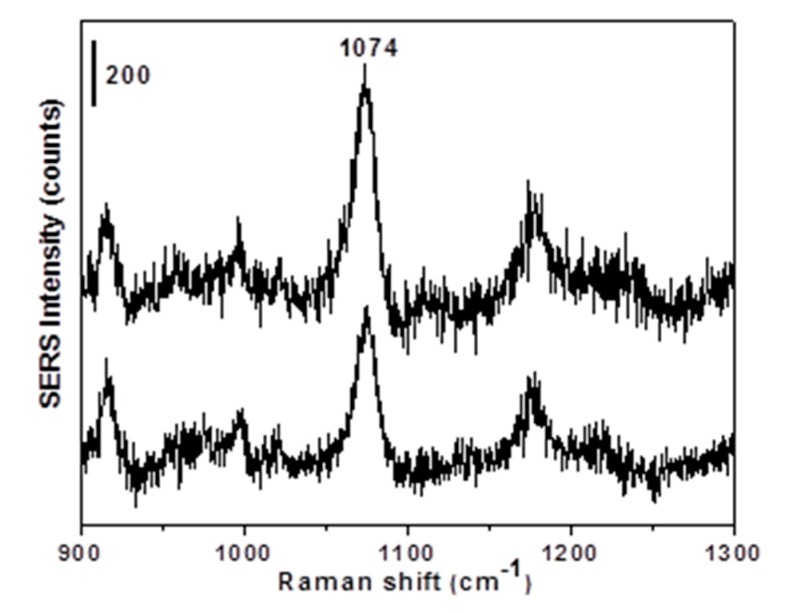
SERS spectra of *p*-MBA after SDME microextraction from a 10^−8^ mol L^−1^ solution. The SERS intensity scale bar is 200 counts. Laser wavelength: 633 nm; laser power: 2.5 mW.

In terms of sensitivity, the SERS detection of *p*-MBA at 10^−8^ mol L^−1^ resulted in a very weak signal (Figure 3), which cannot be clearly distinguished from the noise. To overcome this problem, the SDME technique was applied to pre-concentrate p-MBA from the 10^−8^ mol L^−1^ aqueous solution. In SDME, the solvent volume of extraction was an important variable for the extraction efficiency. Moreover, the amount of the analyte extracted into an organic drop was proportional to the drop size at equilibrium. In this work, 2 µL of toluene was chosen as the extraction solvent volume based on the stability of the generated toluene drop in aqueous solution for 5 min at 22 °C, from previous studies [20]. As can be observed in Figure 5, the *p*-MBA Raman signature at 1074 cm^−1^ was observed in the pre-concentrated SERS (SDME-SERS) spectra.

The average SERS intensity of 1295 ± 245 counts was determined considering the dilution from 2 to 10 µL (drop volume - volume inside the syringe) during the SDME procedure. This value is comparable with the average SERS intensity of the p-MBA solution between the concentrations 10^−4^ mol L^−1^ and 10^−5^ mol L^−1^. Moreover, the value of RSD 20%, obtained with the combination of SDME and SERS, perfectly matches the performance of the substrate; thus, the new p-MBA concentration of ~4.6 × 10^−5^ mol L^−1^ can be extrapolated from the calibration curve (Figure 4a). This result showed that SDME worked very well as a pretreatment technique, increasing the concentration of the analyte by three order of magnitude, from 10^−8^ mol L^−1^ to 10^−5^ mol L^−1^. This large improvement in the level of detection was the key issue in analytical studies and sensors, and it has been explored for many other SERS substrates using p-MBA as a probing molecule [21,22]. Although more Raman measurements should be taken to improve the calibration curve and the signal intensity variation, this work demonstrated that an easy coupling of SDME with SERS can also reach nanomolar detection levels, with the advantage of being a much simpler and faster procedure. Moreover, SDME can be easily tailored (type of solvent, temperature, and time of extraction) and applied for detection of an innumerous variety of substances, even when found in complex matrices, being an optimum alternative for detection of analyte at very low concentrations with SERS.

## 4. Conclusions

AuNHAS with a periodicity of 500 nm was fabricated by interference lithography and its SERS performance was investigated using p-MBA as Raman probe molecule. Different concentrations of p-MBA were easily detected by SERS with a good reproducibility (RSD 21%), from 10^−4^ mol L^−1^ up to the concentration of 10^−7^ mol L^−1^. To improve the analytical applicability of SERS, and lower the detection limit of this analyte, the SDME technique was successfully applied for the pre-concentration of the 10^−8^ mol L^−1^ p-MBA solution. In only five minutes, the SDME method allowed us to obtain a solution of analyte almost a thousand times more concentrated, from 10^−8^ to ~4.6 × 10^−5^ mol L^−1^. Moreover, the tailored choice of extraction solvent, time, and temperature can largely improve the detection of specific analytes in dense or rich matrices, greatly expanding the versatility and applicability of SDME-SERS analysis. Ultimately, for the first time, this work demonstrated that the coupling of the SDME technique with SERS analysis is a very promising method for a rapid and easy-to-apply quantitative detection of molecules at very low concentrations.
